# Supplementary material for: Anisotropic carrier dynamics and laser-fabricated luminescent patterns on oriented single-crystal perovskite wafers
Source: Nat Commun. 2024 Jan 30;15:914. doi: 10.1038/s41467-024-45055-y (PMC10828488; doi:10.1038/s41467-024-45055-y)
Supplement: Supplementary file 1 — Supplementary Information [file 41467_2024_45055_MOESM1_ESM.pdf]

Supplementary Information for

Anisotropic Carrier Dynamics and Laser-Fabricated Luminescent Patterns on Oriented Single-Crystal Perovskite Wafers

Chao Ge[1,2†]*, Yachao Li[1,3†], Haiying Song[1]*, Qiyuan Xie[1], Leilei Zhang[4], Xiaoran Ma[1,3], Junfeng Liu[5], Xiangjing Guo[1], Yinzhou Yan[1], Danmin Liu[5], Wenkai Zhang[3]*, Shibing Liu[1], Yang Liu[2]*

Affiliations

[1]Institute of Laser Engineering, School of Physics and Optoelectronic Engineering, Beijing University of Technology, Beijing 100124, China

[2]State Key Laboratory of Crystal Materials, Shandong University, Jinan 250100, China

[3]Department of Physics and Applied Optics Beijing Area Major Laboratory, Center for Advanced Quantum Studies, Beijing Normal University, Beijing 100875, China

[4]State Key Laboratory of NBC Protection for Civilian, Beijing 102205, China

[5]Key Laboratory of Advanced Functional Materials, School of Materials Science and Engineering, Beijing University of Technology, Beijing 100124, China

*Corresponding authors. E-mail: gechao@bjut.edu.cn; hysong@bjut.edu.cn; wkzhang@bnu.edu.cn; liuyangicm@sdu.edu.cn

[†] These authors contributed equally to this work.

Table of Contents

Figure S1. Crystalline quality characterizations of $MAPbBr_3$ wafers.

Figure S2. The in-plane and out-of-plane views of $MAPbBr_3$ crystal structure.

Figure S3. Schematic illustration of the angle-resolved transient transmission apparatus.

Figure S4. Pseudo colour TA plots of $MAPbBr_3$ with 400 nm excitation and 515 nm excitation.

Figure S5. Schematic illustration of the transmission open-aperture Z-scan technique.

Figure S6. The SEM images of femtosecond laser processed scalable patterns on (001), (100) and (111) $MAPbBr_3$ wafers.

Figure S7. Effect of processing scanning speed on luminescence.

Figure S8. Pseudo colour TA plot of $MAPbBr_3$ (100) wafer is plotted by logarithmic delay time.

Figure S9. The relaxation dynamics of free carriers and excitons before and after laser processing.

Figure S10. The analyzation of photogenerated excitons dynamics.

Figure S11. Anisotropic relaxation dynamics evolution of $MAPbBr_3$ probed at 575 nm.

Figure S12. Dielectric function anisotropy of differently oriented $MAPbBr_3$ single-crystal wafers.

Figure S13. The intensity-dependent PLQYs of $MAPbBr_3$ crystals before and after processing as a function of charge density.

Figure S14. Pseudo colour TA plots and TA spectra on untreated $MAPbBr_3$ wafer at different excitation densities.

Figure S15. TA spectra at different excitation densities at 1.5 ps of processed $MAPbBr_3$ crystals.

Table S1. Pump fluence dependence fitting for carrier relaxation dynamics in $MAPbBr_3$ (100) wafer.

Table S2. Probe polarization dependence fitting for carrier relaxation dynamics in $MAPbBr_3$ (100) wafer.

Table S3. Probe polarization dependence fitting for carrier relaxation dynamics in $MAPbBr_3$ (110) wafer.

Table S4. Probe polarization dependence fitting for carrier relaxation dynamics in $MAPbBr_3$ (111) wafer.

Supplementary Note 1. The analyzation of photogenerated excitons dynamics.

Supplementary Note 2. Anisotropic relaxation dynamics evolution of free carriers in $MAPbBr_3$.

Supplementary Note 3. The long-range Fröhlich interaction in $MAPbBr_3$.

Supplementary Note 4. The analyzation of correlation of polarization-dependent dynamics to the crystal structure.

Supplementary Note 5. The analyzation of intensity-dependent PLQYs and TA spectra.

Supplementary References

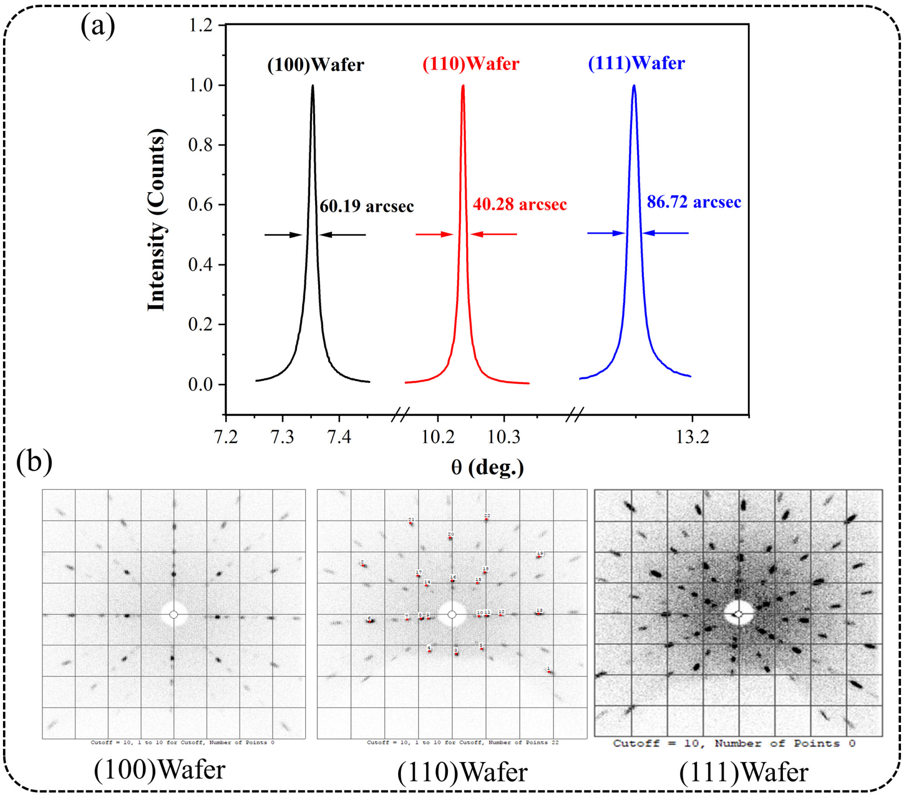

Figure S1. Crystalline quality characterizations of $MAPbBr_3$ wafers. (a) High-resolution X-ray diffraction rocking curves of $MAPbBr_3$ wafers on their respective (100), (110), and (111) planes. **(b)** Laue diffractions of $MAPbBr_3$ wafers on their respective (100), (110) and (111) planes.

In order to prove the high quality of the grown $MAPbBr_3$ crystal, we have performed high-resolution X-ray diffraction (HRXRD) on the three single crystal wafers. **Figure S1a** shows the rocking curves in a θ scan mode. We can see the $MAPbBr_3$ crystal wafers show an FWHM (full width at half-maximum) of 60.19 arcsec for (100), 40.28 arcsec for (110), and 86.72 arcsec for (111). As we all known that the FWHM of HRXRD or the X-ray diffraction rocking curve is an important index to evaluate crystalline quality of single crystals, wherein a perfect crystal produces a symmetrical and sharp peak profile with a small FWHM. Here our measured FWHM are much smaller than the reported values for $MAPbBr_3$ crystals[1-3], and is even comparable with those of the well-developed inorganic crystals[4], demonstrating the grown crystals possessing high crystalline perfection. Furthermore, we have also measured the X-ray Laue diffraction patterns of the $MAPbBr_3$ wafers. As shown in **Figure S1b**, the well-defined laue diffractions further confirm the high crystal quality of the $MAPbBr_3$ wafers.

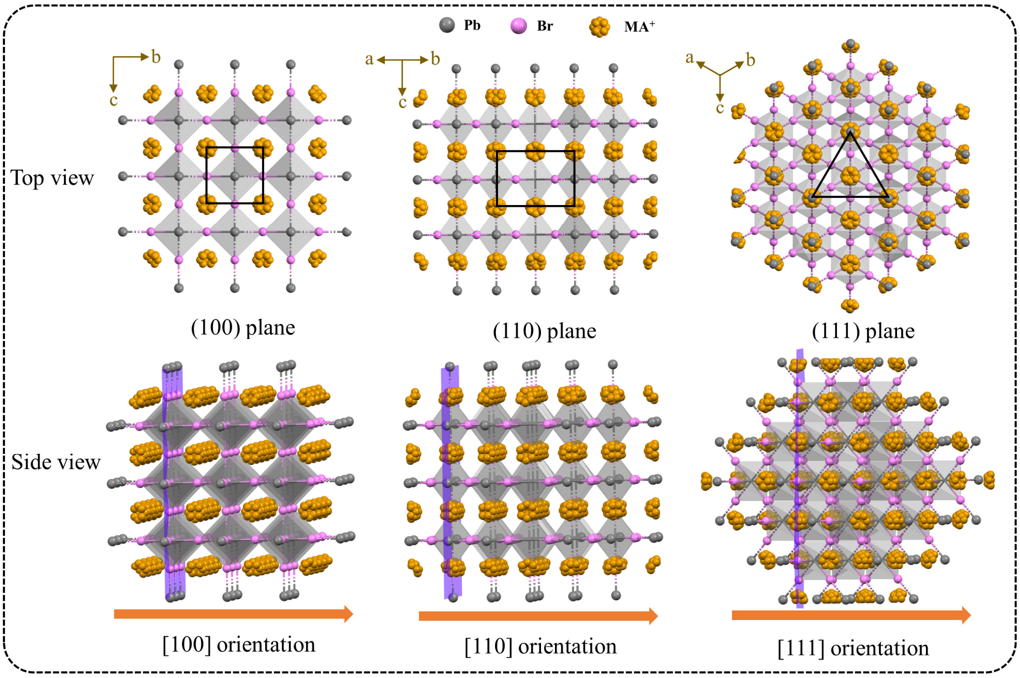

Figure S2. **The in-plane and out-of-plane views of $MAPbBr_3$ crystal structure with different orientations.** The in-plane (top) views of the $MAPbBr_3$ crystal structure reveal two-dimensional arrangements forming a continuous network. The out-of-plane (side) views of the MAPbBr3 crystal structure display the stacking of (100), (110), and (111) planes, respectively, resulting in three-dimensional arrangements.

At room temperature, $MAPbBr_3$ adopts a cubic $Pm\bar{3}m$ space group based on X-ray crystallography statistics. The crystal structure of $MAPbBr_3$ consists of an inorganic framework of corner-sharing $PbBr_6$ octahedra and organic MA^+ cations. The MA^+ cations are located in the interstitial spaces between the $PbBr_6$ octahedra, which are assumed to be randomly oriented in a spherical manner to satisfy the O_h point group. The arrangement of the anions and cations, the atom density and the interplanar spacing can vary depending on the crystal orientation. Here is a brief description of the crystal structure for different orientations in **Figure S2**:

<u>(100) wafer</u>: In this wafer, the crystal planes are vertical to the [100] orientation. The crystallographic geometric configuration of the (100) plane exhibits 4-fold rotational symmetry.

<u>(110) wafer</u>: In this wafer, the crystal planes are vertical to the [110] orientation. The crystallographic geometric configuration of the (110) plane exhibits 2-fold rotational symmetry.

<u>(111) wafer</u>: In this wafer, the crystal planes are vertical to the [111] orientation. Compared to the [100] and [110] orientations in $MAPbBr_3$, the [111] orientation achieves the highest packing density. The crystallographic geometric configuration of the (111) plane exhibits 3-fold rotational symmetry.

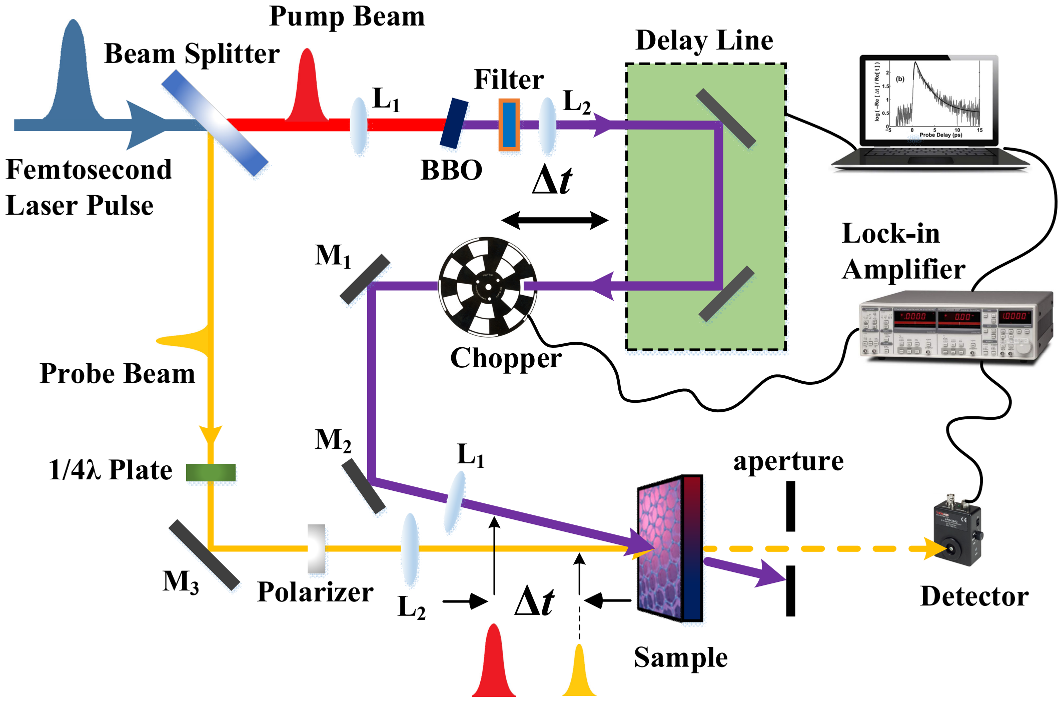

Figure S3. Schematic illustration of the angle-resolved transient transmission measurement based on ultrafast time-resolved pump-probe technique. The polarization of the probe light is modulated by a 1/4 λ wave plate and a polarizer.

Table S1. Pump fluence-dependent transient transmittance with pump and probe polarization fixed along 0° and 90°. The $|\Delta T/T_0|$ of the $MAPbBr_3$ (001) wafer is fitted by a double-exponential model. The decay parameters of carrier relaxation dynamics are tabulated in **Table S1.**

Fitting Function	$\Delta T/T_0 = A_1$*exp($-t/\tau_1$) + A_2*exp($-t/\tau_2$) + A_0					
Pump Fluence (μJ/cm^2)	71	142	213	284	355	426
A_1	4.84522E-5 ± 8.42105E-7	1.1273E-4 ± 1.34055E-6	1.77473E-4 ± 1.69408E-6	2.49837E-4 ± 2.41161E-6	3.17259E-4 ± 3.00205E-6	3.34575E-4 ± 2.71281E-6
τ_1 (ps)	14.83228 ± 0.49197	8.62977 ± 0.2055	6.70164 ± 0.12147	5.17426 ± 0.08929	4.93 ± 0.08264	5.05273 ± 0.07347
A_2	3.83089E-5 ± 7.40399E-7	6.47769E-5 ± 9.59491E-7	8.65664E-5 ± 9.57498E-7	1.06843E-4 ± 1.1553E-6	1.2565E-4 ± 1.41204E-6	1.31599E-4 ± 1.32792E-6
τ_2 (ps)	105.19739 ± 3.09251	74.89678 ± 1.43611	68.87816 ± 1.01044	57.7027 ± 0.78406	55.11021 ± 0.76497	54.91648 ± 0.6767

Table S2. Probe polarization dependence fitting for carrier relaxation dynamics in $MAPbBr_3$ (100) wafer. (pump laser fluence: 71 μJ/cm^2)

Fitting Function	$\Delta T/T_0 = A_1$*exp($-t/\tau_1$) + A_2*exp($-t/\tau_2$) + A_0							
Polarization	0°	15°	30°	60°	120°	150°	165°	180°
A_1	7.2048E-5 ± 1.97632E-6	3.5567E-5 ± 1.03273E-6	3.7847E-5 ± 1.09701E-6	2.5256E-5 ± 1.27872E-6	2.5235E-5 ± 1.07597E-6	3.7847E-5 ± 3.97675E-6	3.5567E-5 ± 2.05784E-6	8.0007E-5 ± 1.17381E-6
τ_1 (ps)	14.0012 ± 0.32934	8.0809 ± 0.45067	4.8295 ± 0.42119	4.2432 ± 0.63425	4.2352 ± 0.48635	4.8295 ± 0.38642	8.0809 ± 0.78842	13.928 ± 0.55661
A_2	4.6097E-5 ± 8.37473E-7	3.004E-5 ± 7.07383E-7	2.3597E-5 ± 6.80884E-7	1.8085E-5 ± 9.68453E-7	1.8076E-5 ± 5.99884E-7	2.3597E-5 ± 7.27026E-7	3.004E-5 ± 7.55944E-7	3.7474E-5 ± 8.20683E-7
τ_2 (ps)	116.2476 ± 3.38474	92.9825 ± 5.16539	87.8235 ± 4.68596	68.9952 ± 8.62745	68.9924 ± 7.08953	87.8257 ± 4.89247	92.9858 ± 12.01956	115.2447 ± 5.67865

Table S3. Probe polarization dependence fitting for carrier relaxation dynamics in $MAPbBr_3$ (110) wafer. (pump laser fluence: 71 μJ/cm^2)

Fitting Function	$\Delta T/T_0 = A_1$*exp($-t/\tau_1$) + A_2*exp($-t/\tau_2$) + A_0							
Polarization	0°	45°	90°	135°	180°	225°	270°	315°
A_1	1.0053E-4 ± 3.8756E-6	1.14681E-4 ± 5.18882E-6	1.14137E-4 ± 5.92581E-6	1.04129E-4 ± 6.6789E-6	1.0343E-4 ± 7.00502E-6	1.11756E-4 ± 1.04812E-5	1.07003E-4 ± 1.17891E-5	1.16032E-4 ± 1.1798E-5
τ_1 (ps)	6.72274 ± 0.33702	6.85274 ± 0.3108	6.61291 ± 0.33241	6.79207 ± 0.21803	6.94306 ± 0.50863	6.76792 ± 0.41433	6.54351 ± 0.35064	6.67874 ± 0.32694
A_2	2.00474E-5 ± 9.29897E-7	2.12277E-5 ± 1.06459E-6	2.04683E-5 ± 1.05788E-6	1.85936E-5 ± 7.78636E-7	1.94785E-5 ± 1.33657E-6	1.9728E-5 ± 1.48011E-6	2.01666E-5 ± 1.32997E-6	1.83313E-5 ± 1.19156E-6
τ_2 (ps)	85.21401 ± 6.1192	87.25635 ± 5.08631	83.12434 ± 6.00969	86.42331 ± 4.629	82.87642 ± 10.50368	87.13354 ± 8.61594	84.31272 ± 6.41169	88.32177 ± 8.04558

Table S4. Probe polarization dependence fitting for carrier relaxation dynamics in $MAPbBr_3$ (111) wafer. (pump laser fluence: 71 μJ/cm^2)

Fitting Function	$\Delta T/T_0 = A_1$*exp($-t/\tau_1$) + A_2*exp($-t/\tau_2$) + A_0								
Polarization	0°	15°	30°	60°	90°	135°	150°	165°	180°
A_1	4.0884E-5 ± 2.71828E-6	3.6723E-5 ± 1.96143E-6	1.9757E-5 ± 2.54963E-6	2.8919E-5 ± 2.13805E-6	2.3313E-5 ± 1.78655E-6	2.9884E-5 ± 2.05173E-6	2.5281E-5 ± 2.62371E-6	3.2678E-5 ± 2.07646E-6	4.4969E-5 ± 2.57421E-6
τ_1 (ps)	11.0012 ± 0.62306	10.2182 ± 0.77969	8.5668 ± 1.52384	6.7205 ± 1.10726	7.4085 ± 1.62286	7.0256 ± 0.90435	9.4647 ± 1.35111	10.9964 ± 1.12919	10.8177 ± 0.87957
A_2	2.4415E-5 ± 1.23715E-6	1.4938E-5 ± 1.15786E-6	2.2855E-5 ± 1.93386E-6	1.4885E-5 ± 8.79473E-7	1.6698E-5 ± 1.45519E-6	1.9051E-5 ± 9.91409E-7	1.8682E-5 ± 1.62644E-6	1.5565E-5 ± 1.18487E-6	1.5929E-5 ± 1.17194E-6
τ_2 (ps)	125.49632 ± 7.39257	94.54635 ± 10.79514	85.2072 ± 4.85921	86.69317 ± 15.35244	78.55145 ± 15.49168	85.58077 ± 13.11174	82.49376 ± 8.58629	103.9589 ± 14.42511	124.09893 ± 11.97071

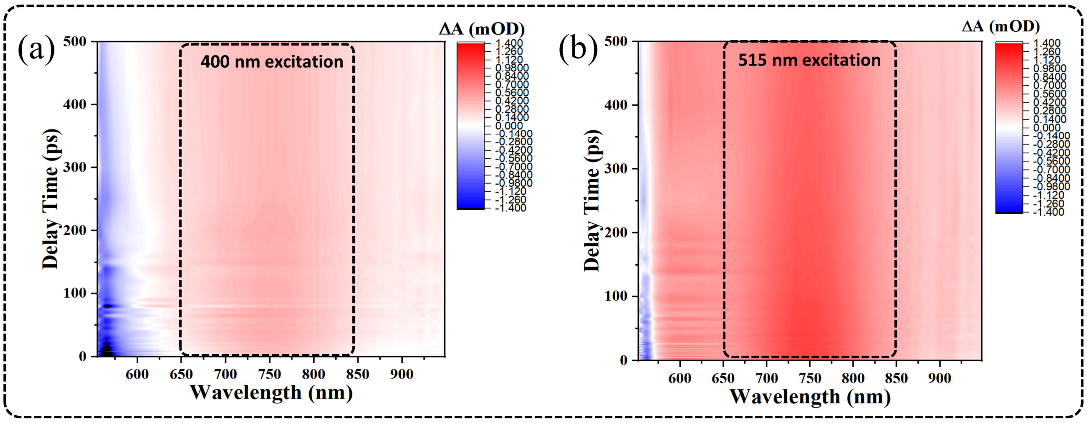

Figure S4. Pseudo colour TA plots of $MAPbBr_3$ with 400 nm excitation (a) and 515 nm excitation (b).

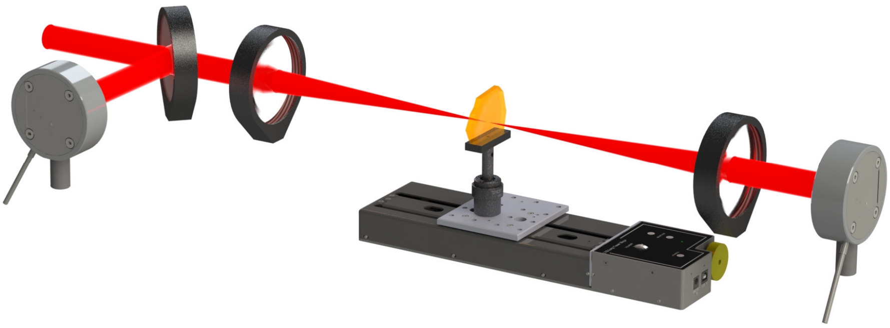

Figure S5. Schematic illustration of the transmission open-aperture *Z*-scan technique. The experiment is carried by moving the sample back-and-forth from the focal point of the pulsed laser beam along its optical axis (defined as the *z*-axis).

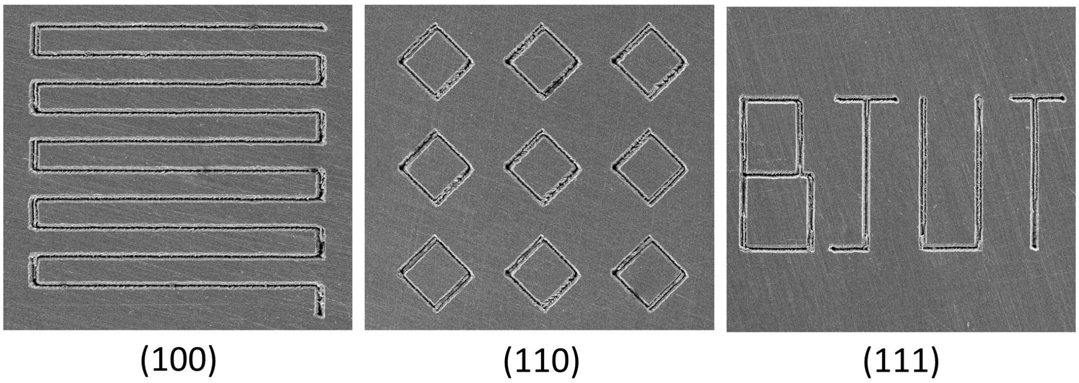

Figure S6. The scanning electron microscope (SEM) images of femtosecond laser processed scalable patterns on three $MAPbBr_3$ wafers. [laser power: 5 mW, defocus: 470 μm and scanning speed: 0.1 mm s^{-1}]

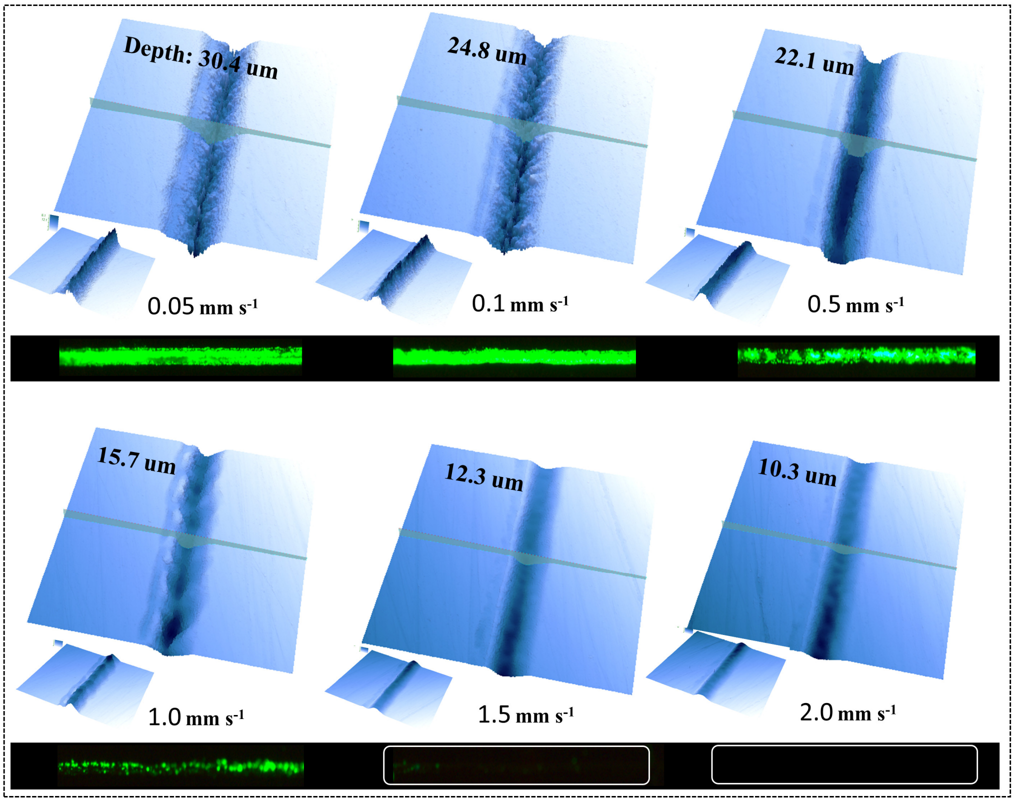

Figure S7. Effect of processing scanning speed on luminescence. The front-view and back-view colored 3D profile images of LSCM and corresponding fluorescent microscopy (FM) images at different processing scanning speeds indicate that the scanning speed of 0.1 mm s^{-1} corresponds to the strongest fluorescence emission. [laser power: 5 mW, defocus: 470 μm]

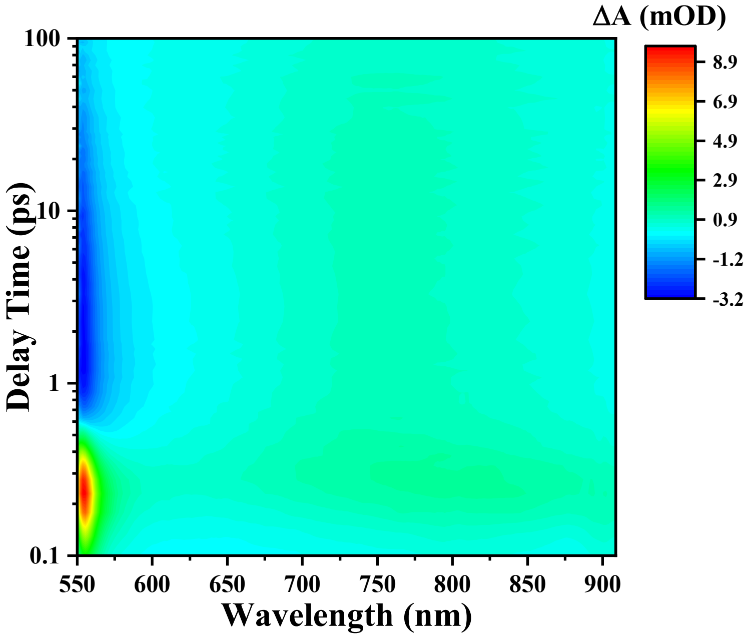

Figure S8. Pseudo colour TA plot of $MAPbBr_3$ (100) wafer is plotted by logarithmic delay time. Prior to the appearance of the bleach signal, the light-induced absorption signal observed within 600 femtoseconds appears to be an unavoidable coherent artifact present in bulk single

crystals (with the thickness in millimeters and above), which is attributed to the non-degenerate two-photon absorption (TPA) when the pump and probe beams overlap both temporally and spatially.

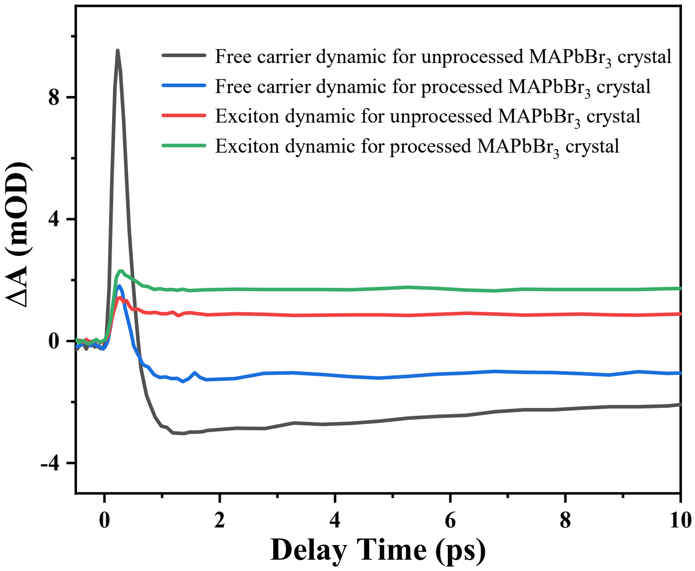

Figure S9. The relaxation dynamics of free carriers and excitons were investigated before and after laser processing, with probing wavelengths of 555 nm and 800 nm, respectively.

Supplementary Notes

Supplementary Note 1. *The analyzation of photogenerated excitons dynamics*

It has been reported that there should be two kinds of primary photoexcitations in the hybrid perovskites, namely free carriers in the valence band (VB) and/or conduction band (CB); and excitons which are Coulomb correlated electron-hole (e-h) bound pairs. The reported exciton binding energy of $MAPbBr_3$ perovskite ranges from 40 to 150 meV,[5-7] larger than the thermal energy (~26 meV) at room temperature. Therefore, the photogenerated excitons and free carriers are expected to coexist according to Saha–Langmuir equation.[8-10] ("*with typical excitation densities of around* 10^{16}*–*10^{17} cm^{-3} *for PL quenching experiments, free carrier population is found to be in the range of ≈50%–90% when trap states are not considered*." Tze Chien Sum et al., *Adv. Energy Mater.* **2016**, 1600551) Additionally, according to the reference suggested by the reviewer below (*Nat. Photonics* **2015**, *9*, 695), it is reported that the electrostatic potential variations in smaller polycrystals suppress exciton formation, while the larger single-crystals of the same composition demonstrate an unambiguous excitonic state.

In our work the relaxation dynamics of the excited-carriers in the picoseconds timescale was monitored by using a polarized probe pulse at 1.55 eV, following an above-bandgap pump

at 3.1 eV (the energy bandgap of $MAPbBr_3$ single crystal is ~2.3 eV) with low excitation density (1.4×10^{15} cm^{-3}). According to the TA spectra (**Figure S10a**), there is observed an obvious positive ΔA signal, signifying the occurrence of photoinduced absorption (PA) in the broad spectral region of λ >600 nm (**Figure S10b**). The PA signal became stronger as the excitation density increased (**Figure S10c**), which confirms that it should mainly originate from the excited hot carriers. Here, we ascribe the negative ΔA signals close to the optical gap as the ground-state bleaching (GSB), which is attributed to state-filling by excited carriers. However, it is evident that the dynamic of PA is different from that of the bleaching band, as shown in the **Figure S10d**, indicating that these two bands originate from different photoexcitations. We therefore assign the PA band to reflect dynamics of the photogenerated excitons. The observation is consistent with the investigation by Anita Ho-Baillie et al. [*J. Phys. Chem. C* **2016**, *120*, 2542] that "*The much faster rise of PA_2 indicates a different origin from bleaching, which is most likely to be the absorption of photogenerated excitons for several reasons.*" Sheng et al. [*PRL* **2015** *114,* 116601] also attributed a similar absorption band after photoexcitation of $MAPbPbI_3$ to generation of exciton. When assuming the PA band around ~ 750 nm was attributed to exciton absorption, it's important to relate the exciton PA to specific transitions between distinct excitonic states. For $MAPbBr_3$, the E_I and E_{III} exciton states, separated by an energy of ~1.642 eV [*Solid State Commun.* **2003**, *127,* 619], coincidently corresponds to the PA band at ~750 nm (~1.650 eV) in the TAS. Hence, it is reasonable to speculate that the PA band is attributed to the inter-band exciton absorbing transition (E_I to E_{III}).

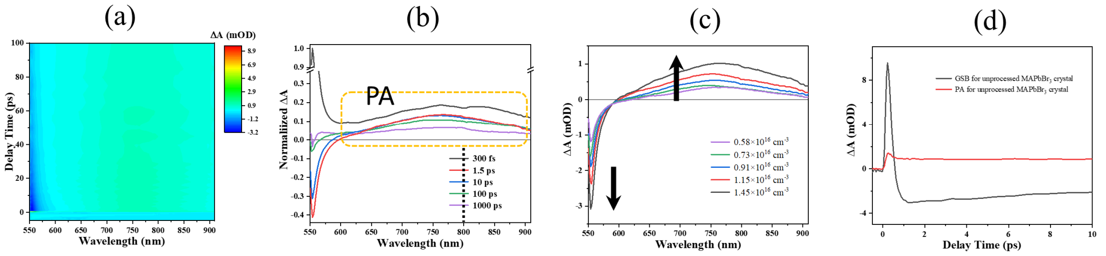

Figure S10. The analyzation of photogenerated excitons dynamics. (a) Pseudo colour TA plot with the excitation density of 1.45×10^{16} cm^{-3}. **(b)** TA spectrum at different delay time. **(c)** TA spectrum at different excitation densities at 1.5 ps. **(d)** The ground-state bleaching (GSB) and photoinduced absorption (PA) dynamics are probed at 555 nm and 800 nm, respectively.

However, the authors of [*Adv. Energy Mater.* ***2016****, 6, 1600551*] considered the PA band "*possibly arises from the transitions of the photoexcited species to higher excited states or sub-bandgap trap state absorption in the crystal, which is significant in the very thick crystal (≈ 3.2 mm) while negligible in the thin film (≈100 nm)*"; and the authors of [*Adv. Optical Mater.* ***2018****, 6, 1700975*] concluded that "*While we cannot eliminate all other mechanisms, we believe that polaron formation is the most likely hypothesis for our TA observation in perovskite bulk single crystals.*" The above reports demonstrate that there is indeed divergence on the assignment of the PA band. Here given the low amplitude and longer lifetime of the PA band, and the "soft" lattice nature of $MAPbBr_3$ crystal, it is more likely that free carriers, excitons, and polarons coexist in the photogenerated states.

Whatever, at present all the assignments of the longer wavelength PA band are short of direct and solid evidence. In fact, even the exact band structures and the actual fundamental

band-gap values of such hybrid perovskites are still under intense debate in the computational community. [*J. Phys. Chem. Lett.* **2017**, *8,* 5507; *J. Chem. Theory. Comput.* **2016**, *12,* 3523] To quantitatively assign the features in the TA spectra to specific transitions through theoretical calculations is infeasible. Thus although it is considered that exciton absorption is a most likely hypothesis for the observed longer wavelength PA in perovskite bulk single crystals, we cannot eliminate the possibilities from other attributions such as polarons or other sub-bandgap trap state absorption. And the predictability of the specific transitions necessitates more detailed experimental and theoretical investigations in this field.

Supplementary Note 2. *Anisotropic relaxation dynamics evolution of free carriers in* $MAPbBr_3$

The polarization anisotropy detected at the wavelength of 800 nm, which may predominantly reflect the selection rules between excitonic states rather than those of free carriers. Therefore, based on the transient absorption spectra of $MAPbBr_3$ crystals (**Figure S4**), we measured the dynamics at a wavelength of 575 nm that located on the bleaching band, to better capture the relaxation processes of free carriers.

The carrier dynamics probing at 575 nm on differently-oriented $MAPbBr_3$ wafers were conducted with the same test conditions as that in 800 nm detections (a 3.1 eV femtosecond pump laser and a pump fluence of 71 μJ cm^{-2}). The bleaching dynamics at 575 nm (shown in the insets of **Figure S11**) can also be fitted well by two shorter lifetimes: τ_1 within ~ 20 ps and τ_2 within ~ 150 ps, and another longer lifetime, τ_3, being fitted at the nanosecond scale. The overall time scale of the bleaching dynamics is slightly longer than that of the PA dynamics, but the time of peak occurrence is almost identical to the PA band, both around 1.5 ps, corresponding to the electron-electron scattering and electron-longitudinal optical (LO) phonon scattering.

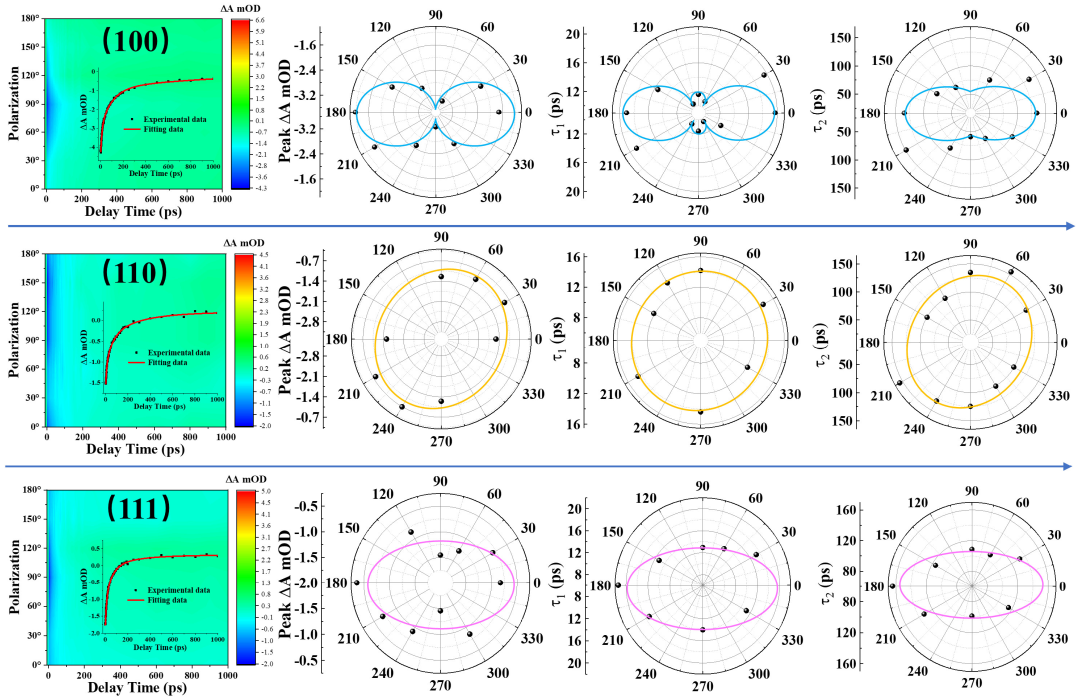

Figure S11. Anisotropic relaxation dynamics evolution of $MAPbBr_3$ probed at 575 nm.

Figure S11 shows the pseudo-color polarization-resolved transient absorption plots of the bleaching dynamics at 575 nm on the (100), (110) and (111) wafers. As can be seen for each stage of the carrier dynamics, including the extremely fast cooling stage represented by the peak value of ΔA, and the two decay stages represented by the lifetimes (τ_1 and τ_2), they indeed demonstrated obvious angle-dependence upon variation of the probe polarizations. Compared to the anisotropy of dynamics probed at 800 nm, the anisotropic behaviors probed at 575 nm shows similar polarization-dependent symmetry, with a milder contrast among different orientations. Compared to free carriers, excitons should possess relative more localized nature in crystal, thus stronger interactions with the lattice and phonons during the relaxation process should be anticipated, which makes them more susceptible to the influence of the lattice structure. On the one hand, the additional subtle variations may be caused by inevitable light disturbances owing to the close proximity of signals at different polarization angles during measurements.

Overall, the dynamics probing at the beaching band of 575 nm should reflect more information about free carriers of the excited state of $MAPbBr_3$. Although there are some differences in the relaxation lifetimes and polarization-dependent symmetry patterns, both the dynamics of free carriers and excitons exhibit obvious in-plane polarization dependence on (100) and (111) wafers while a weak dependence on the (110) wafer. Because the relaxation processes for both the two types of particles involve interactions with lattice vibrations (phonons) in the decay to lower energy levels or the ground state, they are influenced by the intrinsic factors such as the symmetry of electronic band structure, phonon spectra, and other lattice-related elements. Therefore, similar selection rules may take effect during the relaxation process regardless of the particle type.

Supplementary Note 3. *The long-range Fröhlich interaction in $MAPbBr_3$*

As we know, the Fröhlich model addresses the electrons in ionic crystals or polar semiconductors.[12] The strength of the Fröhlich interaction in a material is directly linked to the polar nature of its crystal lattice. In a highly polar material the Coulomb field of a carrier (or exciton) couples more easily to the polar vibrations (*i.e.* LO phonons) of the lattice, resulting in strong Fröhlich coupling. In the structure of $MAPbBr_3$ crystal, it consists of two interpenetrating sublattices: an inorganic lattice composed of corner-shared $PbBr_6^{4-}$ octahedral and a second sublattice composed of MA^+ cations. The non-superimposed arrangement of the positive and negative charges leads to the existence of electric dipoles in the lattice and makes the perovskite lattice polar. Due to the polar nature of $MAPbBr_3$, Fröhlich interaction is the dominant relaxation pathway for hot carriers, wherein the electron-longitudinal optical (LO) phonon scattering arises from Coulomb interactions between the electrons and the macroscopic electric field induced by LO phonon mode. Thus the Fröhlich interaction that governs electron-lattice coupling is considered to be long-range interaction. And it has been widely accepted that the Fröhlich interaction is crucial in describing carrier behavior in the polar lattice of lead halide perovskites.[13–16]

The electron-phonon interactions lead to the formation of a polaron state, where an electron or a hole deforms the lattice in its vicinity and becomes more localized.[17] To date, the

conventional Fröhlich interaction has been mainly considered to be the polaron formation mechanism in halide perovskites. Depending on the range and strength of the electron−phonon interaction, polarons can be generally categorized into large and small polarons. For the 3D lead halide perovskites, large polarons are formed by the long-range electron-LO phonon interaction, *i.e.*, the charge carriers coupling to the vibrational motion of the inorganic lattice, while the A site cation only plays a minor and indirect role by affecting the distortion of the inorganic lattice. (Conversely, a small polaron is formed by strong and short-range electron−phonon interactions and is usually localized within a single lattice constant. Small polarons are mostly revealed in the low-dimensional halide perovskites and double perovskites. The Fröhlich interaction model is inapplicable to the strong electron−phonon coupling of small polarons.)

The long-range nature of Fröhlich interaction has been confirmed through several experimental and theoretical evidences.[18-20] In particular, Zhu et al.[21] have provided a direct time domain view of large polaron formation in single-crystal $MAPbBr_3$ and $CsPbBr_3$ using time-resolved optical Kerr effect (TR-OKE) spectroscopy and in conjunction with hybrid density functional theory (DFT) calculations, obtaining the electron and hole polaron mobilities of μ_e = 149.8 $cm^2\ V^{-1}\ s^{-1}$ and μ_h = 79.2 $cm^2\ V^{-1}\ s^{-1}$ with corresponding polaron radii of ρ_e = 4.18 nm and ρ_h = 3.13 nm in $MAPbBr_3$. Lindenberg et al.[22] visualized excitation-induced strain fields in $MAPbBr_3$ via femtosecond resolution diffuse X-ray scattering measurements, confirming the formation of large polarons with a polaron radius of ~3 nm at t = 20 ps. Consequently, the observation of large polarons, which extend over more than a few lattice sites, provides further evidence supporting the existence of long-range Fröhlich interaction in $MAPbBr_3$ crystals.

About whether the interaction strength will differ along different planes, because the electron−phonon scattering relates to carrier effective mass, LO phonon energy, and dielectric constant, we could find the dependence between crystallographic planes and the strength of electron-phonon coupling interaction according to the so-called Fröhlich constant α is defined by,[23]

$$\alpha = \frac{e^2}{\hbar\omega_0}\left(\frac{1}{\varepsilon_\infty} - \frac{1}{\varepsilon_S}\right)\sqrt{\frac{m\omega_0}{2\hbar}}$$

Where ε_∞ and ε_s are optical and static dielectric constants, respectively, m is the effective mass of the electron in the case of no electron-phonon interaction (bare band mass), and ω_0 describes the LO phonon frequency.

The quantity $\frac{1}{\bar{\varepsilon}} = \left(\frac{1}{\varepsilon_\infty} - \frac{1}{\varepsilon_S}\right)$, which is known as the dielectric contrast, quantifies the ionic nature of a material and thereby determines the strength of the carrier–lattice interaction. Consequently, the static and high-frequency values of the dielectric function provide a means of evaluating the Fröhlich interaction among differently oriented planes.

Thus we analyzed the dielectric function of differently oriented $MAPbBr_3$ wafers by measurements of the refractive index (k) and the extinction coefficient (n) through a

spectroscopic ellipsometry (SE) method. (**Figure S12a & b**) The complex refractive index, $N = n - ik$, contains the same information as the dielectric function, $\varepsilon = \varepsilon_1 - i\varepsilon_2$, with $\varepsilon_1 = n^2 - k^2$ and $\varepsilon_2 = 2nk$.[24] By analysis of n and k as a function of wavelength, it is notable that for both real (ε_1) and imaginary (ε_2) dielectric function in the optical region, the values indeed show discrepancies for differently oriented wafers. (**Figure S12c & d**) Deducing from the dielectric mechanism, the dielectric contrast should be even larger in the low-frequency THz region where the LO phonons typically reside. Therefore, on account of the measured $\varepsilon_{1(110)} > \varepsilon_{1\ (100)} > \varepsilon_{1\ (111)}$, the difference of dielectric function on different crystal planes will definitely affect the Fröhlich interaction strength along different planes.

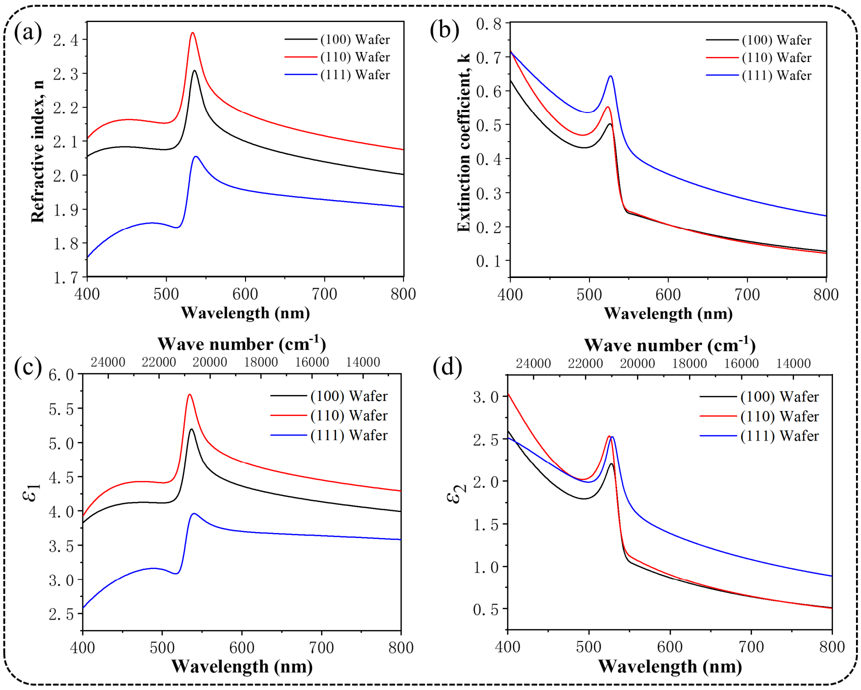

Figure S12. Dielectric function anisotropy of differently oriented $MAPbBr_3$ single-crystal wafers. (a) Refractive index (n), **(b)** extinction coefficient (k), **(c)** real (ε_1) and **(d)** imaginary (ε_2) part of the dielectric function on (100), (110), and (111) oriented wafers.

Supplementary Note 4. *The analyzation of correlation of polarization-dependent dynamics to the crystal structure*

The in-plane polarization-dependent dynamics on the (100) and (111) wafers and the isotropic dynamics on the (110) wafer represent one of the most prominent discovery revealed by this study. In the current experiment, the relaxation time scale mainly concerns the carrier dynamics involving hot carrier cooling, polaron formation, exciton formation, and the subsequent dynamics of "cold" carriers near the band-edge involving photoinduced lattice expansion, strain, and coherent phonon effects. During these photophysical processes, the carrier−phonon interaction plays a significant role by inducing local lattice displacements which generate a polarization-induced electric field that in turn interacts with the charge carriers. Thus the involvement of phonons (lattice vibrations) in all these stages corelates the carrier relaxation dynamics with the lattice structure.

From a structural view, although $MAPbBr_3$ is regarded as a highly symmetric cubic structure at room temperature resting on identification of the organic MA^+ cation as having random orientations in the X-ray crystallography statistics,[25] our group and other researchers have indeed revealed in-plane and out-of-plane structure anisotropy through angle-resolved Raman spectroscopy, as well as orientation-dependent optoelectronic properties by using photoconductivity and photoluminescence measurements.[26-29] These recent investigations unambiguously corroborated the steady-state anisotropy on $MAPbBr_3$ single crystals, however, the transient-state anisotropy in the ultrafast carrier dynamics still remains sealed. This is because the anisotropy of steady-state properties relies directly on the static crystal structure that we can depict intuitively; while for the ultrafast carrier dynamics occurring in the excited state, there are two main obstacles. Firstly, photoexcitation can lead to a distinct excited electronic structure compared to the ground state (so far, there is still a big challenge to accurately capture the excited electronic structures experimentally). Secondly, the carrier relaxation process is heavily influenced by phonons, which are hard to describe with a clear picture. Therefore, it is infeasible to directly corelate the carrier relaxation dynamics with the electronic structure of the ground state. In spite of these challenges, we can still find clues from analyzing the distortion of the $PbBr_6$ octahedral framework and the dynamic orientation of MA^+ cations. Additionally, investigating the defect density distribution along different crystallographic orientations and considering the surface and many-body effects can provide further understanding. These factors may affect the excited electronic structure and phonon behavior of $MAPbBr_3$ single crystal.

Lattice deformation, vibration and relaxation effect: Because of the different crystallographic geometric configurations of the (100), (110) and (111) planes (**Figure S1**, including the atomic density, interplanar spacing, etc.) and the distribution of MA^+ orientation domains, the equilibrium out-of-plane and in-plane anisotropic structure should induce non-synchronized lattice deformation and relaxation upon photoexcitation, which may break the original lattice symmetry in the excited state structure. E.g., the MA^+ rocking and twisting vibration modes demonstrate distinct in-plane anisotropy on (100) and (110) wafers, which may in turn cause interactions on the distortion of the $PbBr_6^{4-}$ inorganic skeletons; and the transient photoluminescence lifetimes also show discrepancies among (100), (110) and (111) wafers.[26] Consequently the incoherent lattice vibration amplitude and frequency dictate the specific phonon modes along different crystallographic orientations in $MAPbBr_3$. Thus an azimuthally balanced lattice deformation on the (110) plane is imagined to induce a polarization-independent excited state dynamics on the (110) crystal plane.

Trap density distribution and surface effect: Because on the different crystallographic planes, the defect formation energies of vacancies and interstitials are different; and likewise, the ion migration also depends on the crystallographic orientations, these will lead trap density distributions inhomogeneously on different crystal wafers. By using space charge limited current (SCLC) measurements performed on (100), (110) and (111) wafers, we have demonstrated the discrepancy of trap densities on differently oriented wafers.[27] Carrier scattering and trapping resulting from defects play a crucial role in influencing carrier relaxation in different orientations within the bulk crystal. Additionally, the polaronic nature of the relatively soft lattice in $MAPbBr_3$ introduces surface dipoles that affect electronic structure

and charge distribution. These factors also become significant determinants of carrier transport and recombination properties on distinct crystal planes. Thus a more highly symmetrical distribution of the excited-state charge density on the (110) crystal plane is anticipated.

Many-body effect: The interactions among electrons and with other particles, such as phonons (lattice vibrations) and other carriers, can lead to many-body effects. These effects cause a redistribution of momentum and energy of the excited state electrons through energy exchange or resonance coupling between the particles. Many-body effect has significant consequences on the carrier's lifetime and optical properties such as absorption and refraction in the excited state, thus making the carrier dynamics even more complicated.

Therefore, due to the uncomparable excited state electronic structure with that of the ground state, the mechanisms behind ultrafast hot carrier relaxation process are too subtle to be accurately learned. Frankly speaking, we still do not have exact answers about why $MAPbBr_3$ (110) wafer behaves distinctly in the excited-state carrier relaxations compared to other wafers. It is important to note that it requires more detailed experimental investigation and deeper analysis to correlate the observed polarization-dependent dynamics with the crystallographic structures in different crystal planes. This study, based on the differently-oriented $MAPbBr_3$ single-crystal wafers, firstly penetrated into the orientation-dependent dynamical evolution of the excited carriers and provided solid observations of the anisotropy in the ultrafast carrier dynamics. Although a comprehensive understanding has not been achieved yet due to limitations in excited-state experimental techniques, this discovery holds significant implications as it provides a novel perspective for a deeper understanding of the ultrafast carrier relaxation pathways and opens up new possibilities for utilizing perovskite single crystals in polarization-sensitive photoelectron responses.

Supplementary Note 5. *The analyzation of intensity-dependent PLQYs and TA spectra*

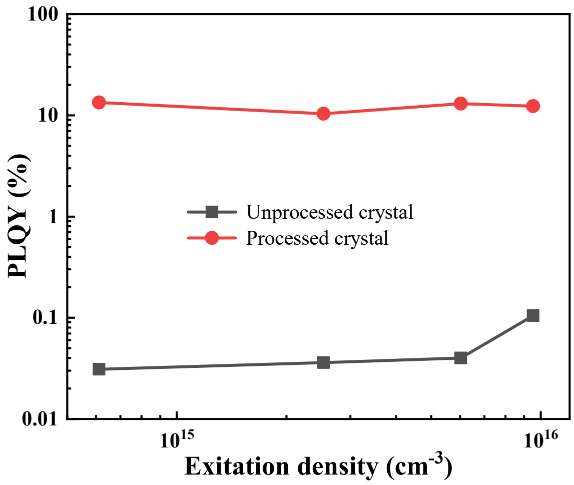

Figure S13. The intensity-dependent PLQYs of $MAPbBr_3$ crystals before and after processing as a function of charge density.

In **Figure S13**, we show the experimental steady-state PLQY data as a function of excitation density of $MAPbBr_3$ crystals before and after processing, respectively. We note that

the pristine $MAPbBr_3$ (unprocessed bulk crystal) exhibits a very low PLQY of <0.1% under the measurement conditions with excitation densities lower than 10^{16} cm^{-3}. While the processed crystal shows a much higher quantum yield in the order of ten percent at similar densities (and under ambient atmosphere). We find that the PLQY of the pristine $MAPbBr_3$ crystal shows an upward change at high excitation fluences. This trend is consistent with the model described in ref [*Phys. Rev. Appl.* **2014**, *2*, 034007] that the charge-trapping pathways limit the radiative recombination at low excitation fluences, and the radiation would be significantly enhanced when the charge trap states are filled by photogenerated carriers at higher excitation fluences. Additionally, we have confirmed the presence of metallic Pb(0) defect in the pristine bulk $MAPbBr_3$ and its transformation into oxygen passivated-Pb(II) after laser processing by X-ray photoelectron spectroscopy (XPS). Because the excess Pb(0) atoms have been proved to act as deep defect levels that cause nonradiative decay to degrade the photoluminescence, it is suggested that its transformation into the oxygen passivated-Pb(II) corresponds to a transition from nonradiative deep-level traps to radiative shallow traps after laser processing. According to the model described in ref [*J. Am. Chem. Soc.* **2016**, *138*, 13604], the participation of shallow defects in the radiative process leads to an increased PLQY compared to the case of deep trap states. Our PLQY measurements show a pronounced increase from the pristine crystal to the laser-processed one, indicating the formation of shallow-trap states by induction of femtosecond laser and/or passivation of the deep-level traps by ambient oxygen. Due to the limit of the power of excitation light source, the excitation intensity-dependence of the measured PLQYs for both the pristine and processed $MAPbBr_3$ remain pretty insensitive at the lower excitation intensity range (<10^{16} cm^{-3}). However, a slight enhancement of the PLQY was still observed on the pristine crystal at higher excitation intensity. This slight increase of PLQY may be attributed to the increased filling of trap states and, possibly, to an increasing excitonic fraction of photogenerated species at higher excitation intensity. [*Phys. Rev. Appl.* **2014**, *2*, 034007] It's reported the substantial increase of PLQY for the case of shallow traps may occur at even higher excitation intensity due to the high depopulation rate R_{dep} of the trapped carriers compared to that of the deep traps.[*J. Am. Chem. Soc.* **2016**, *138*, 13604] It is noted that due to the fact that the measured PLQY of the pristine crystal is quite low, even within the degree of measurement error, the conclusion is indeed lack of solid evidences.

The involvement of different types of defects is also supported by the intensity-dependent TAS measurements. **Figure S14** illustrates that in the pristine crystals, the intensities of both the photoinduced bleach (PB) bands and the photoinduced absorption (PA) bands (exciton absorption band) exhibit an upward trend as the excitation density rises; on the other hand, for the laser processed $MAPbBr_3$ crystal, the PA bands intensity remains nearly constant as rising of the excitation density. (**Figure S15**) Furthermore, considering the phenomenon we observed in the measurements that the pristine crystals only showed perceptible green emissions under very strong laser excitation, it indicates that deep-level defects dominate the recombination in the pristine bulk crystals. These deep-level defects need more photogenerated carriers to fill up, thus the signals of the exciton absorption become prominent under stronger excitation. While in the laser processed crystal, the exciton fraction from the photogenerated free carriers is high even under low excitation density because there is no such a pre-process filling. And because

of the competition from radiative recombination via the shallow traps, the population of the photogenerated excitons may keep in a relatively steady level with increasing of the excitation fluences. This observation is further corroborated by the redshift in the bleaching band compared to the pristine crystal. The authors of [*JPCC* ***2016****, 120, 2542*] regarded it "*The extension of the bleaching signal at longer wavelength indicates the presence of shallow trap state.*" Overall, the creation of shallow-trap states induced by femtosecond laser and/or transformed from passivation of the deep traps by ambient oxygen are considered to be one of the key factors responsible for the observed enhancement in PL.

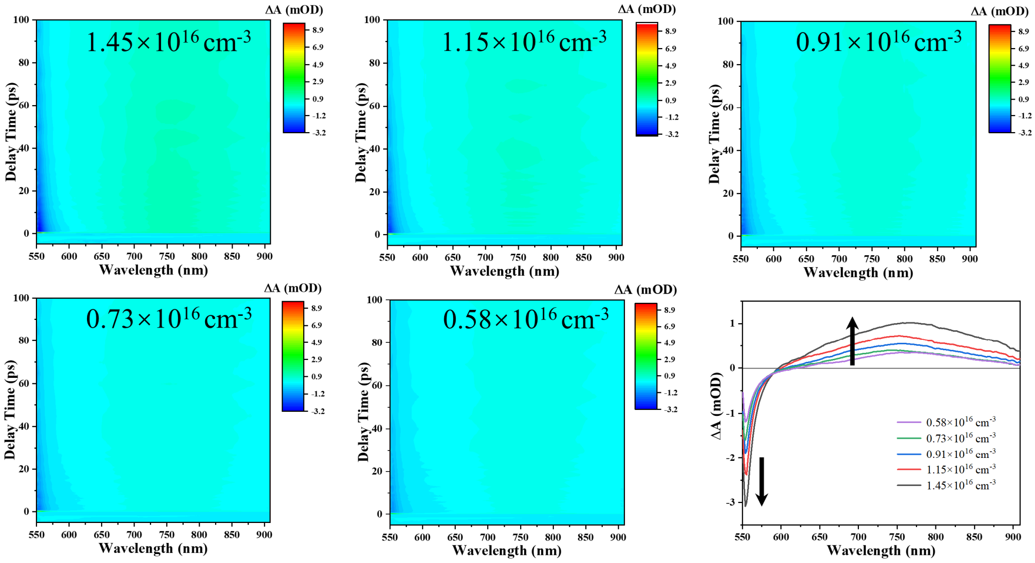

Figure S14. Pseudo colour TA plots and TA spectra (at delayed 1.5 ps) on untreated $MAPbBr_3$ wafer at different excitation densities.

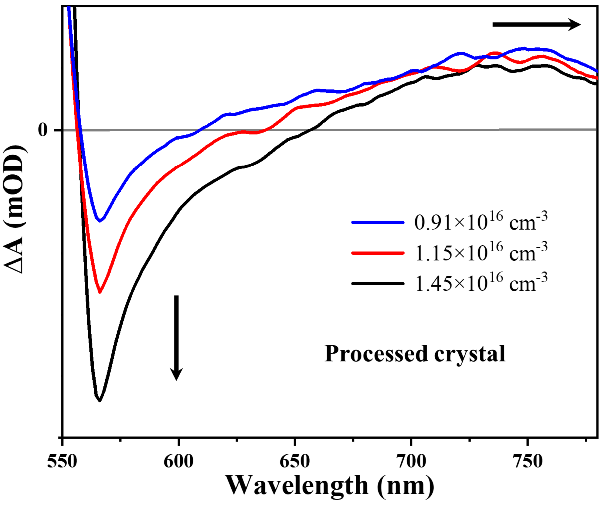

Figure S15. TA spectra at different excitation densities at 1.5 ps of processed $MAPbBr_3$ crystals.

Supplementary References

1 Jing L, Cheng X, Yuan Y, et al. Design growth of triangular pyramid MAPbBr3 single crystal and its photoelectric anisotropy between (100) and (111) facets [J]. *The Journal of Physical Chemistry C*, **2019**, *123*(17): 10826-10830.

2 Su J, Sang L, Wang D, et al. Solution growth and morphology of CH3NH3PbBr3 single crystals in different solvents [J]. *Crystal Research and Technology*, **2016**, *51*(11): 650-655.

3 Peng W, Wang L, Murali B, et al. Solution-grown monocrystalline hybrid perovskite films for hole-transporter-free solar cells [J]. *Advanced Materials*, **2016**, *28*(17): 3383-3390.

4 Hu Q, Jia Z, Volpi A, et al. Crystal growth and spectral broadening of a promising Yb: CaLu x Gd 1− x AlO 4 disordered crystal for ultrafast laser application [J]. *CrystEngComm*, **2017**, *19*(12): 1643-1647.

5 Tanaka K, Takahashi T, Ban T, et al. Comparative study on the excitons in lead-halide-based perovskite-type crystals CH3NH3PbBr3 CH3NH3PbI3 [J]. *Solid state communications*, **2003**, *127*(9-10): 619-623.

6 Koutselas I B, Ducasse L, Papavassiliou G C. Electronic properties of three-and low-dimensional semiconducting materials with Pb halide and Sn halide units [J]. *Journal of Physics: Condensed Matter*, **1996**, *8*(9): 1217.

7 Yang Y, Yang M, Li Z, et al. Comparison of recombination dynamics in CH3NH3PbBr3 and CH3NH3PbI3 perovskite films: influence of exciton binding energy [J]. *The journal of physical chemistry letters*, **2015**, *6*(23): 4688-4692.

8 Sestu N, Cadelano M, Sarritzu V, et al. Absorption F-sum rule for the exciton binding energy in methylammonium lead halide perovskites [J]. *The journal of physical chemistry letters*, **2015**, *6*(22): 4566-4572.

9 Saba M, Cadelano M, Marongiu D, et al. Correlated electron–hole plasma in organometal perovskites [J]. *Nature communications*, **2014**, *5*(1): 5049.

10 D'innocenzo V, Grancini G, Alcocer M J P, et al. Excitons versus free charges in organo-lead tri-halide perovskites [J]. *Nature communications*, **2014**, *5*(1): 3586.

11 J. I. Pankove, Optical Processes in semiconductors, Dover Books, Dover, NY **1971**.

12 Yamada Y, Kanemitsu Y. Electron-phonon interactions in halide perovskites [J]. *NPG Asia Materials*, **2022**, *14*(1): 48.

13 Frost J M, Whalley L D, Walsh A. Slow cooling of hot polarons in halide perovskite solar cells [J]. *ACS energy letters*, **2017**, *2*(12): 2647-2652.

14 Iaru C M, Geuchies J J, Koenraad P M, et al. Strong carrier–phonon coupling in lead halide perovskite nanocrystals [J]. *ACS nano*, **2017**, *11*(11): 11024-11030.

15 Zhu X Y, Podzorov V. Charge carriers in hybrid organic–inorganic lead halide perovskites might be protected as large polarons [J]. *The Journal of Physical Chemistry Letters*, **2015**, *6*(23): 4758-4761.

16 Li M, Bhaumik S, Goh T W, et al. Slow cooling and highly efficient extraction of hot carriers in colloidal perovskite nanocrystals [J]. *Nature communications*, **2017**, *8*(1): 14350.

17 Zheng F, Wang L. Large polaron formation and its effect on electron transport in hybrid perovskites [J]. *Energy & Environmental Science*, **2019**, *12*(4): 1219-1230.

18 Quarti C, Grancini G, Mosconi E, et al. The Raman spectrum of the CH3NH3PbI3 hybrid perovskite: interplay of theory and experiment [J]. *The journal of physical chemistry letters*, **2013**, *5*(2): 279-284.

19 La-o-Vorakiat C, Xia H, Kadro J, et al. Phonon mode transformation across the orthohombic–tetragonal phase transition in a lead iodide perovskite CH3NH3PbI3: a terahertz time-domain spectroscopy approach [J]. *The journal of physical chemistry letters*, **2016**, *7*(1): 1-6.

20 Leguy A M A, Goñi A R, Frost J M, et al. Dynamic disorder, phonon lifetimes, and the assignment of modes to the vibrational spectra of methylammonium lead halide perovskites [J]. *Physical Chemistry Chemical Physics*, **2016**, *18*(39): 27051-27066.

21 Miyata K, Meggiolaro D, Trinh M T, et al. Large polarons in lead halide perovskites [J]. *Science advances*, **2017**, *3*(8): e1701217.

22 Guzelturk B, Winkler T, Van de Goor T W J, et al. Visualization of dynamic polaronic strain fields in hybrid lead halide perovskites [J]. *Nature materials*, **2021**, *20*(5): 618-623.

23 Devreese J T. Polarons[M]//Encyclopedia of applied physics: vol. 14. **1996**: 383-409.

24 Löper P, Stuckelberger M, Niesen B, et al. Complex refractive index spectra of CH3NH3PbI3 perovskite thin films determined by spectroscopic ellipsometry and spectrophotometry [J]. *The journal of physical chemistry letters*, **2015**, *6*(1): 66-71.

25 Nandi P, Pandey S K, Giri C, et al. Probing the electronic structure of hybrid perovskites in the orientationally disordered cubic phase [J]. *The Journal of Physical Chemistry Letters*, **2020**, *11*(14): 5719-5727.

26 Zhang L, Cui S, Guo Q, et al. Anisotropic Performance of High-Quality MAPbBr3 Single-Crystal Wafers [J]. *ACS Applied Materials & Interfaces*, **2020**, *12*(46): 51616-51627.

27 Zhang L, Liu Y, Ye X, et al. Exploring anisotropy on oriented wafers of MAPbBr3 crystals grown by controlled antisolvent diffusion [J]. *Crystal Growth & Design*, **2018**, *18*(11): 6652-6660.

28 Zuo Z, Ding J, Zhao Y, et al. Enhanced optoelectronic performance on the (110) lattice plane of an MAPbBr3 single crystal [J]. *The Journal of Physical Chemistry Letters*, **2017**, *8*(3): 684-689.

29 Yang H, Zhou Y, Yang Y, et al. Crystal facet engineering induced anisotropic transport of charge carriers in a perovskite [J]. *Journal of Materials Chemistry C*, **2018**, *6*(43): 11707-11713.
